# Announcement

**DOI:** 10.1111/acel.70599

**Published:** 2026-06-22

## 1 Editorial Leadership Transition at *Aging Cell*

The Anatomical Society and Wiley would like to inform the *Aging Cell* community that Monty Montano will be stepping down from his role as Editor‐in‐Chief.

We extend our sincere thanks to Monty for his leadership and commitment over the past 3 years. During his tenure, he has played an important role in developing the journal, supporting the community, and strengthening *Aging Cell*'s position within the wider field of geroscience. We wish him every success in his future endeavors.

## 2 A Collaborative Approach to the Future of *Aging Cell*

We will be introducing a new editorial structure designed to reflect the breadth of the field, support the journal's continued development, and ensure strong, consistent editorial oversight. This structure aims to balance strategic leadership with subject‐specific expertise while maintaining the high standards expected by the community.

The new structure will comprise four new Co‐Editors‐in‐Chief, who will collectively provide shared strategic leadership and oversight of editorial standards for *Aging Cell*. They will be supported by a team of Associate Editors to manage the peer review process and secure the journal's speciality expertise across the field, giving the journal the flexibility to expand and evolve with the dynamics of the community. In addition, the journal will be appointing a Reviews and Special Issues Editor, focusing on the development of high‐quality invited manuscripts and thematic collections. The Editorial Advisory Board will continue to provide valuable subject expertise, community insight, and strategic input to support the journal's direction.

The new editorial structure has been developed as part of a close collaboration between Wiley and the Anatomical Society to ensure that *Aging Cell* continues to serve as a trusted platform for high‐quality research across the scope of aging biology. We are grateful to the *Aging Cell* community for its continued support and engagement, and look forward to working together with the new editorial team in this next phase of the journal's development.

